# The Influence of Porosity on Mechanical Properties of PUR-Based Composites: Experimentally Derived Mathematical Approach

**DOI:** 10.3390/polym15081960

**Published:** 2023-04-20

**Authors:** Miroslav Černý, Josef Petruš, Ivana Chamradová

**Affiliations:** 1 Faculty of Chemistry, Institute of Materials Science, Brno University of Technology, Purkyňova 464/118, 612 00 Brno, Czech Republic; miro.ce@centrum.cz (M.Č.); josef.petrus@ceitec.vutbr.cz (J.P.); 2 Central European Institute of Technology, Brno University of Technology, Purkyňova 656/123, 612 00 Brno, Czech Republic

**Keywords:** polymer matrix composite, mechanical properties, porosity

## Abstract

The work is focused on the mechanical behavior description of porous filled composites that is not based on simulations or exact physical models, including different assumptions and simplifications with further comparison with real behavior of materials with different extents of accordance. The proposed process begins by measurement and further fitting of data by spatial exponential function *z_c_ = z_m_* · *p*_1_^*b*^ · *p*_2_^*c*^, where *z_c_*/*z_m_* is mechanical property value for composite/nonporous matrix, *p*_1_/*p*_2_ are suitable dimensionless structural parameters (equal to 1 for nonporous matrix) and *b*/*c* are exponents ensuring the best fitting. The fitting is followed by interpolation of *b* and *c*, which are logarithmic variables based on the observed mechanical property value of nonporous matrix with additions of further properties of matrix in some cases. The work is dedicated to the utilization of further suitable pairs of structural parameters to one pair published earlier. The proposed mathematical approach was demonstrated for PUR/rubber composites with a wide range of rubber filling, various porosity, and different polyurethane matrices. The mechanical properties derived from tensile testing included elastic modulus, ultimate strength and strain, and energy need for ultimate strain achievement. The proposed relationships between structure/composition and mechanical behavior seem to be suitable for materials containing randomly shaped filler particles and voids and, therefore, could be universal (and also hold materials with less complicated microstructure) after potential following and more exact research.

## 1. Introduction

Porous materials are commonly presented in our surroundings as different foams, ceramics, concrete, etc. Therefore, they can have one component or composite nature. It is not easy to describe the relationship between mechanical properties, structure, and composition and then predict the behavior of real porous material. Many researchers have studied and described the mentioned problem. They used various materials in their investigations. However, each study was done on materials with slow variation in the composition, typically one kind of porous metal with different degrees of porosity [1,2,3,4,5], ceramics [1,4,6,7,8,9,10,11,12,13,14], polymers [1,15], and natural materials [16]. In all cases, elastic modulus and/or strength were studied. The functions include different mathematical forms. They are linear [1,5,6,7,8,9,10,11,12,16], exponential [1,5,6,11,12], power [1,2,3,4,6,9,11,12,14,15,17,18,19], and logarithmic [5]. Dependencies are mainly empirical or represent some models based on some physical assumptions requiring limits in simplification in the material structure. The dependencies are usually quite simple with some fitting parameters [1,10,20,21].

Composite materials are more complex in their compositions and structures. Two approaches can be used for solving this problem—microscopic and macroscopic [22]. The microscopic approach is based on the knowledge or assumptions of the microstructure of the material. It has the form of models [23,24,25,26,27,28] and/or numerical simulations represented by fractals utilization [23], finite elements method [29,30], and fast Fourier Transform [31]. Models often include the rule of mixture [23,25,28,29]. Microscopic approach is accurate in the physical and mechanical description and the structure description of materials. However, these approaches work on the assumption of different behaviors of ideal and real material structures. There are usually differences between assumptions and real behavior of material due to differences between ideal and real structure. This approach is often found in the literature [23,24,25,26,27,29,30,31,32] despite mentioned limitations. Differences in the comparison of real and predicted results are evident in many works [23,27,28,29,31] concerned with the elastic modulus or the elastic region of the loading as well as in works with prediction of nonlinear stress [24,25,26,30,32]. There are also various structural assumptions, such as spherical void shape [27], granular particle shape [32], cylindrical shapes of fibers without any contact with voids [29], nanopores and circular cross-sections of unidirectional nanofibers [31], particles without contact to voids and discretization to domains containing different parts of filler particles distribution [23] and filling of nanotubes or nanoparticles [25,27,31], a two-phase system where one phase is separated by second phase (in the form of surface layer) from open porosity [30].

The macroscopic approach can be based on the rules of mixture corresponding to weight average content and properties of material components to obtain the composite material behavior [22]. It is less complex in the used level of mathematics and does not explain the accurate physical behavior on the microstructure level of material. On the other hand, it is not limited by some ideal structure of materials and is also suitable for the description of materials embodying no-regular structure in the form of void/particle shape and placement. Accordingly, it could be more useful for the general description of complex materials with no more complicated simple microstructure. Instead of the microstructural approach, there are no predictions confirmed by real behavior with some extent of success, but the measurement is at the beginning, and then the relationships describing the material are found by obtaining the best match between the real results and results based on knowledge of material components properties. Our composite material contains irregular particles and voids, therefore, we have chosen the macroscopic approach for our research. The macroscopic approach was used in our previous work [33] based on the same primary data as this new one. The macroscopic point of view is also applied in work dedicated to tensile testing of porous composites filled by nanofibers describing the fracture probability according to fracture toughness and porosity of material by Weibull analysis [34].

The macroscopic approach is a great challenge for complex systems such as porous composites. There is no offer of parameters set directly and completely describing the material behavior according to one general equation per property. As published elsewhere [33], the behavior of composites has to be fitted according to suitable structural parameters generating the equation(s) containing parameters clearly connected with the composition/structure of the material and fitting parameters. These fitting parameters are chosen according to the best fitting without a strict connection to the material behavior. However, they can be connected with the component behavior of composite materials to make partial equations. Therefore, the fitting parameters are very important because they give the meaning to the whole equation(s) [33], unlike the fitting number without exact meaning and equations for only one-component materials [1,10,20,21] before the work [33] was published.

The novelty of the proposed topic is in agreement with many scientific papers published within the last decade and dedicated to various porous composites differing by composition and also potential applications. The study of mechanical behavior is usually included, however, without any general and exact description of the relationship between mechanical behavior and structure and composition. We can mention some examples as regenerated cellulose + cross-linked poly (ethylene glycol) for potential biomedical applications, packaging, and sewage purification with a higher rate of porosity reaching 97% [35], properties of glass-ceramic binder doped by nanocopper [36] alloy of titanium, aluminum, and vanadium with the addition of silver particles for biomedical applications up to 50% of porosity [37], even combination of ceramics and epoxy resin or polycaprolactone with approximately 70% of porosity [38].

Spatial exponential function containing suitable structural parameters (interspace filling and interspace volume—*n_p_* and 1 − *v_f_*) and exponents as the fitting members were used in our previous study [33]. This new study extends it. It uses the same primary data and shares the same approach with it. The novelty is an enlargement of obtained results including new combinations of structural parameters in relationships and further dependencies describing the new cases of fitting parameters. The basic equation shape is retained and is described in more detail in the following parts of this work. Higher count of used structural parameters indicates further possibilities of research in this field and can favorably influence further research.

## 2. Materials and Methods

### 2.1. Materials

Polyurethane matrices were based on isocyanate pre-polymer Unixin 4223CS (Lear, Brno, Czech Republic). The pre-polymer was a mixture of condensed based on methylene-di-phenyl-di-isocyanate (MDI) with 6.9 wt.% of NCO groups, number average molecular weight of 690 g/mol, density of 1.10 g/cm^3^, and viscosity of 2800 ± 500 mPa·s. The pure uncondensed MDI created 10–30 wt.% of isocyanate mixture of pre-polymer. Distilled water, castor oil (Fichema, Brno, Czech Republic), and glycerol (Penta Chemicals, Prague, Czech Republic) acted as curing agents. Linseed oil (Fichema, Brno, Czech Republic) acted as a plasticizer. Di-butyl-tin-di-laureate (DBTL, Lear, Brno, Czech Republic) was used for acceleration of curing. The chemistry of curing reactions is depicted in Figure S1 in Supplementary Materials.

Rubber filler was produced by grinding car tires and was supplied by RPG Recycling, Uherský Brod, Czech Republic. Three fractions of ground rubber differing in particle size distribution were labeled as R_0_, R_1_, and R_2_ in this work. The numbers in subscripts are ordered according to particle sizes in fractions distributions when higher number means larger particle sizes in distribution. R_1_ and R_2_ fractions were obtained by knife milling and R_0_ was obtained by milling by rolling machine utilization. Limestone, iron (Pkchemie, Třebíč, Czech Republic), and quartz (Millisil W12, Provodínské Písky, Provodín, Czech Republic) represented inorganic filler and acted as polyurethane matrix modifiers. Iron contained 14–16 wt.% of silicon, according to the supplier. Particle size distribution of inorganic and rubbery filler (except of R_2_) was also determined by laser analyzer HELOS (H2568) & RODOS (Sympatec, Clausthall-Zellerfeld, Germany). R_2_ rubber particle size distribution was obtained by sieve analysis due to the size of particles above 800 μm instead of laser analysis inappropriate for such large particles. The shape of filler particles was observed by Scanning electron microscopy (inorganic fillers by TESCAN MIRA 3 (TESCAN ORSAY HOLDING, Brno, Czech Republic) and rubber by Carl Zeiss EVO LS 10 (Carl Zeiss, Oberkochen, Germany)). The density of all fillers was measured using a pycnometer with resulting values (averages from 3 measurements) 1.18 g/cm^3^ for all rubber fractions, 2.65 g/cm^3^ for quartz, 2.68 g/cm^3^ for limestone, and 7.03 g/cm^3^ for iron.

### 2.2. Sample Preparation

Polyurethane matrices were prepared by the mixing of liquid components, including isocyanate pre-polymer, curing agents, di-butyl-tin-di-laureate (DBTL) serving as an accelerator, and the linseed oil serving as a plasticizer if it was a part of the matrix. The solid part consisting of ground rubber or its mixture with inorganic filler was added into the liquid mixture consisting of pre-polymer and curing agents in the next step. The solid part was then mixed with the liquid part. Mixing was carried out manually in all steps. Rubber fractions (R_0_ − R_2_) were not mixed. In each sample, there was only one rubber fraction. The resulting mixture was then loaded into the stainless-steel molds, covered by low-density polyethylene foil for easy handling with specimens. The inner mold dimensions were 120 × 24 × 12 mm. The sample dimensions arose by evolution of looking for sufficiently high dimensions suppressing the randomness of material behavior (the role of defects). Designation and characteristics of all matrices are summarized in Table 1 and are in accordance with data reported in our previously published paper [33]. The volume fraction of rubber fillers is in the range from 20 to 90 vol% with a 10% increment if the porosity is neglected. There are some exceptions caused by the inability of matrices to accept 90 vol% of filler. In any case, the maximum filler content was 70 vol.% for combinations of P_33_-CO_17_-Si_50_/R_0_ and R_1_ (matrix and filler designations from Table 1). Higher maximum 80 vol% was valid for these combinations: P_33_-CO_17_-Si_50_/R_2_, P_72_-G_18_-Ca_10_/R_0_, P_33_-CO_17_-Ca_50_/R_0_ and R_1_ and P_33_-CO_17_-Fe_50_/all rubber filler fractions. The scheme serving as an overview of preparation and testing of samples is depicted in Figure S2.

The chosen polymer system of matrix was used due to simple preparation of plenty of samples which was carried out at room temperature. The second advantage was the possibility of easy modification of one pre-polymer by widely available curing agents to get a lot of different matrices with a common base. The thermoset system is generally unable to create crystals affecting the mechanical behavior (each matrix was the same in all cases, i.e., amorphous). The most significant advantage was the natural creation of porosity by carbon dioxide evolving during curing.

### 2.3. Characterization Methods

The density of filler and P_99_-W_1_ matrix was determined by the pycnometer method. Other matrices (pure PUR, without inorganic filler) with closed porosity were submitted to direct measurement of their weight (8–18 g per sample) and volume. Each measurement was triplicated, and the obtained average value was used for further calculation.

Porosity closed in matrices (except P_99_-W_1_) was evaluated by a confocal laser scanning microscope (Lext OLS 3000, Olympus, Corporation, Shinjuku, Japan). The representative data were obtained from the fracture surface area. Density and porosity of all composites and modified polyurethane matrices (containing inorganic filler) were evaluated from the sample dimensions (measured by digital caliper), weight, and the ratio between real and theoretical density (calculated from densities and ratios of individual components) according to equation:

(1) $$n=1-\frac{\rho }{\rho_{t}}=1-\frac{\frac{m}{V\;}}{\sum v_{it}\cdot \rho_{it}}$$

where *n* is porosity and *ρ* is real density of sample calculated by its weight (*m*) and volume (*V*) given by their dimensions. *ρ_t_* is theoretical density of sample when porosity is neglected, and it is a summation of volume fractions (*v_it_*) and densities (*ρ_it_*) of all components in the sample. The average porosity for each set of samples with the same composition was applied for further calculations of structural parameters (*n_p_* etc.) after the measurement of density of samples and further porosity calculation.

**Figure 1:**
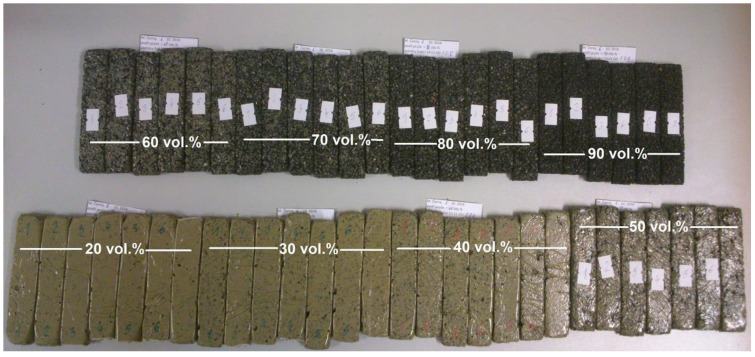
Example of samples before tensile testing. In the figure, there are samples composed of P_33_-CO_17_-Si_50_ matrix and 20–90 vol.% rubber filler R_2_ (if porosity is neglected). The filler content is indicated by white text and attached white lines added to the original figure. Notice: Highly filled specimens in the figure (90 vol.% filler) were not able to be tested due to their nonsolid nature. These specimens are not included in data processing.

Tensile testing of prepared samples was performed by ZWICK Z010 ROELL testing machine (ZWICK ROELL, Ulm, Germany). Dimensions of tested samples (120 × 24 × 12 mm) were chosen sufficiently large to suppress the distinct negative effect of pores on the reproducibility of mechanical properties (see one of samples set in Figure 1). For all measurements, cross-head speed was 30 mm·s^−1^. Observed mechanical properties included elastic modulus (*E*) determined from linear part of tensile curve (strain range from 0.05 to 0.25 %), ultimate tensile strength and strain (*σ_Fmax_* and *ε_Fmax_*), and energy need for ultimate strength achievement (*A_Fmax_*) calculated from the area below the tensile curve according to relationship [32]:

(2) $$A_{F\max}=\sum \left(\varepsilon_{n}-\varepsilon_{n-1}\right)\cdot \frac{\sigma_{n}+\sigma_{n-1}}{2};\;n\;\in \;\langle 1;n_{F\max}\rangle$$

where parameters *σ_n_*, *σ*_*n*−1_, *ε_n_* and *ε*_*n*−1_ represent the strength and strain of neighboring points lying on the tensile curve. *N_Fmax_* is the point of maximum force. Basic unit of the *A_Fmax_* is J·m^−3^ [33]. The other properties linked with the yield point were not studied since the yield point of studied materials was not detected. Five prepared specimens of each sample were tested, and the average value was used for further data processing. The overview of preparation and testing of samples is depicted in Figure S2. Figure S2 also indicates the data output for subsequent data processing.

### 2.4. Basics for Data Processing

We examined the exponential equations from works dedicated to elastic modulus [1,20] and strength [10,21] of one-component material as the base of our proposed system of relationships. Testing of different samples with the same composition and different porosity gives results that can be fitted by exponential or power function. The designations can be different according to the determination of the variable (exponents or powered number). In the further text, we use the designation “exponential”. The equation shape was chosen due to the variability of exponents having potentially positive or negative values enabling easy research work (fitting). Labels in the source Equation (3) [1,10,20,21] are adapted to the abbreviation system used in this article:

(3) $$z_{mn}=z_{m}\cdot {\left(1-n\right)}^{b}$$

where *z_mn_* is the observed mechanical property value for a porous material, *z_m_* is the value of the same mechanical property for a nonporous material, *n* is porosity and *b* is a parameter typical for chosen material (according to several works [1,10,20,21]). However, *b* is not the constant value, but it is a function of *z_m_* as discussed in our previous work (Cerny et al.) [33] focused on porous composites, and all mathematical expressions have a basis in Equation (3). A single structural parameter is insufficient for a system consisting of more than one component. Therefore, the equation valid for composite materials in the work [33] includes two structural parameters for this reason:

(4) $$z_{c}=z_{m}\cdot n_{p}^{b}\cdot {\left(1-v_{f}\right)}^{c}$$

where *z_c_* is an observed value for composite, *n_p_* is interspace filling, 1 − *v_f_* is interspace volume, and exponents *b* and *c* are logarithmic functions with shapes slightly differing according to chosen property. Interspace filling (*n_p_*) is a structural parameter indicating how much the volume among particles is filled by the matrix. It can be calculated with the knowledge of porosity and volume fraction of matrix *v*_*m*(*t*)_ when porosity is neglected. Obtained porosity can be evaluated from the composite composition, density of composite, and densities of individual components—see Equation (1). The expression of *n_p_* is as follows:

(5) $$n_{p}=1-\frac{n}{n+\frac{v_{m(t)}}{1+\frac{n}{1-n}}}$$

The second structural parameter (interspace volume, 1 − *v_f_*) is calculated by Equation (6) containing porosity and interspace filling:

(6) $$1-v_{f}=n\cdot \left(1+\frac{n_{p}}{1-n_{p}}\right)$$

Both parameters are dimensionless and reach values from 0 to 1. It is important to mention the ability of the pair of structural parameters to split the material into the rates of its three components—matrix, filler, and porosity. Exponents *b* and *c* in Equation (4) were interpolated by logarithmic functions to give them real meaning as shown by Equation (7) (elastic modulus *E* is chosen as an example):

(7) $$E_{c}=E_{m}\cdot n_{p}^{d+e\cdot \ln Em}\cdot {\left(1-v_{f}\right)}^{f+g\cdot \ln\left(E_{m}\cdot \delta \right)}$$

where *d*, *e*, *f*, and *g* are number parameters mathematically derived from fitting of experimental data and are essential for chosen filler and *δ* is a parameter related to the polarity of matrix represented by the OH/NCO ratio in polyurethane matrix before curing. All studied properties in that work [33] included elastic modulus as well as the ultimate strength and strain (σ*_Fmax_* and *ε_Fmax_*) and energy need for ultimate strength achievement labeled *A_Fmax_*).

Briefly, previous work [33] was based on PUR-based composite materials composed of only one type of rubber filler and PUR matrices with one pre-polymer type with different compositions ensured by different curing agents and other modifiers, in some cases (see Table 1). Thus, the *δ* values could be used as adhesion parameters with an awareness of simplification. Inorganic fillers used for certain PUR matrices are considered a part of matrix, and the assumption is that the inorganic particles are enclosed in PUR matrix.

The great advantage of Equations (4) and (7) is the possibility of being simplified (porous matrix or nonporous composite). 1 − *v_f_* value is equal to 1 for one-component porous material. Therefore, this member is not part of Equations (4) or (7) if the filler is not presented in the material. The value of *n_p_* is then equal to 1 − *n*. A similar example should be nonporous filled material, where *n_p_* = 1 and Equation (4) is simpler due to only 1 − *v_f_* occurrence.

The relationship between mechanical behavior and structure from the macroscopic point of view (composition + porosity) for filled porous materials is expressed by the pair of structural parameters *n_p_* and 1 − *v_f_*. However, there are also other potential structural parameters that could create pairs capable of fulfilling the same goal as *n_p_* and 1 − *v_f_*. It means each pair of parameters has to give a product equal to 1 for nonporous matrix and product value between 0 and 1 for porous matrix, nonporous, and porous composite, and ideally 0 for filler particles without matrix binding (inability to be loaded by tense). The second important property of the pair of parameters is the ability to describe the composition of material. This means the ability to split the material into filler, porosity, and matrix volume fractions from the values of parameters. The case of *n_p_* and 1 − *v_f_* looks like this: 1 − *v_f_* parameter incorporates the volume fraction of filler in its value, and the *n_p_* parameter value splits the remaining part of the material (interspace) into matrix and porosity. The mentioned parameters, which have to be equal to 1 for nonporous matrix, include *v_m_* (volume fraction of matrix), 1 − *n* (solid rate of material), and *n_pf_* (matrix rate in solid rate of material = matrix volume fraction if the porosity is neglected). These new parameters can be calculated by members occurring in Equations (5) and (6) or their derivatives in Equations (8) and (9). The exception is 1 − *n*, which is only a complementary parameter to porosity.

(8) $$v_{m}=v_{m(t)}\cdot \left(1-n\right)=\left(1-v_{f}\right)\cdot n_{p}$$

(9) $$n_{pf}=\frac{v_{m}}{1-(1-v_{f})+v_{m}}=\frac{v_{m}}{v_{f}+v_{m}}$$

The general goal of this article is to show the other parameter pairs than the *n_p_* and 1 − *v_f_*, if they are suitable for the creation of relationships for the connection of mechanical behavior and structure/composition of porous filled composites and describe the potentially suitable equations. For this purpose, the primary data used for publishing our previous study [33] were used for the proposed extended study. The possibility of more structural parameters and equation forms indicates further possibilities of research in this field.

## 3. Results and Discussion

**Figure 2:**
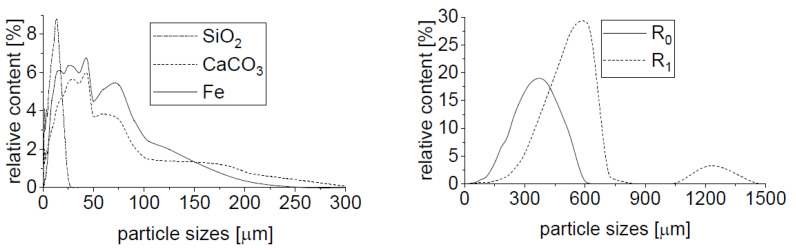
Particle size distribution of used fillers obtained from HELOS (H2568) & RODOS—laser analysis. Figure taken from previous work [33]. Reprinted by permission from Springer Nature Customer Service Centre GmbH: Springer Nature, SN Applied Sciences, A new approach to the structure-properties relationship evaluation for porous polymer composites, Cerny et al., 2020, https://doi.org/10.1007/s42452-020-2479-8, accessed on 22 March 2023, [SN Appl. Sci.], https://www.springer.com/journal/42452, accessed on 22 March 2023.

**Figure 3:**
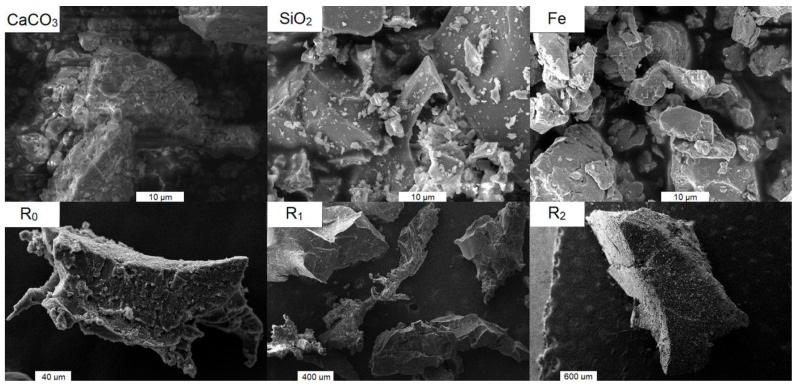
SEM figures depicting particles of used filler obtained by TESCAN MIRA3 (inorganic fillers) and Carl Zeiss EVO LS 10.

**Figure 4:**
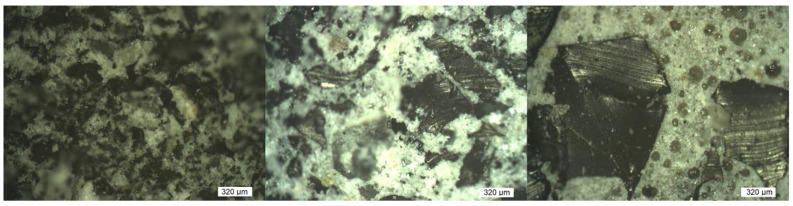
Figures depicting the typical structure of composites in this work created by dark rubber fillers (R_0_, R_1_, and R_2_ from left side to the right side, 40 vol.% in all cases, if porosity is neglected), light matrix P_33_-CO_17_-Ca_50_ matrix (chosen due to good contrast). The pores are visible in the figure containing R_2_ filler and have shapes similar to cubes if they are not in touch with the filler. Figures were observed by optical mode of confocal microscope LEXT OLS 3000.

First, it is necessary to present the properties and roles of individual components before the behavior of composites is described. The main role is played by matrix differences because only one type of filler was considered. The matrix composition is summarized in Table 1 and their density, porosity, and mechanical property values from measurement are given in Table 2. The matrices were chosen to ensure the variety of values of different mechanical properties. The role of filler type is not discussed in this work, although six types of filler were used. There are two reasons for this approach. Three inorganic fillers (limestone, quartz, and iron) were used only as matrix modifiers and are considered to be a part of matrix (see Table 1). The rubber filler (R_0_, R_1_, and R_2_) has the same origin and differs only by their particle size distributions, but the differences in particle sizes are only about one mathematical order, which is insufficient for research of filler difference and its influence on composite behavior. Moreover, the poor differences in fraction sizes distributions enabled the common fitting of the behavior of composites containing different fractions of filler (only one fraction in each sample—fractions were not mixed). The particle size distribution of all inorganic filler and rubber fillers (except R_2_) is depicted in Figure 2. R_2_ rubber distribution was obtained by sieve analysis suitable for particles with a size greater than 800 μm instead of laser analysis. More than 50 wt.% of R_2_ rubber particles have a size greater than 2.5 mm. SEM images of inorganic fillers and rubber filler particles are in Figure 3. The composite structures typical for this work are depicted in Figure 4. There are shown different possibilities of rubber filling (different particle size fractions and the same volume fraction). The matrix P_33_-CO_17_-Ca_50_ containing limestone was chosen due to good contrast with dark rubber filler.

Structural parameters include *n_p_*, 1 − *v_f_*, *v_m_*, 1 − *n* and *n_pf_*. (interspace filling, interspace volume, volume fraction of matrix, solid rate of material and matrix rate in solid rate of material = matrix volume fraction if the porosity is neglected). They are dimensionless, and their values can be in a range from 0 to 1 depending on the composition of the material.

Their values = 1 for nonporous matrices, ≤1 in cases of nonporous filled composite and porous matrix, and <1 in the case of porous filled composite.

Pairs of mentioned structural parameters can be formed as demonstrated for *n_p_* and 1 − *v_f_* in our previous work [33], and each pair of parameters can be used for the description of the composition of the material. It means the ability to determine the volume fraction of filler, matrix and porosity via two parameters. The product of two parameters should be 1 in the case of nonporous matrix and be lower than 1 in the other cases. Then the pair is utilizable for the creation of spatial exponential function (see Equations (4), (10), and (11) as examples). The first fitting of measured properties could have a sufficiently high R^2^ value (coefficient of determination). The last request for a suitable parameter pair is to generate a spatial exponential function with the beginning of the plot (0;0) and have a suitable slope value similar to the real porous matrix (real values available in Table 2). We are aware that this comparison cannot be fully exact, and the suitability of slope value also has an element of estimation (fully nonporous matrices were not available).

The suitable pairs of structural parameters were chosen according to the above-mentioned criteria, and the result is in Table 3. Each pair was eliminated from the further research, so it was clear the pair had not fulfilled some of the required criteria.

**Figure 5:**
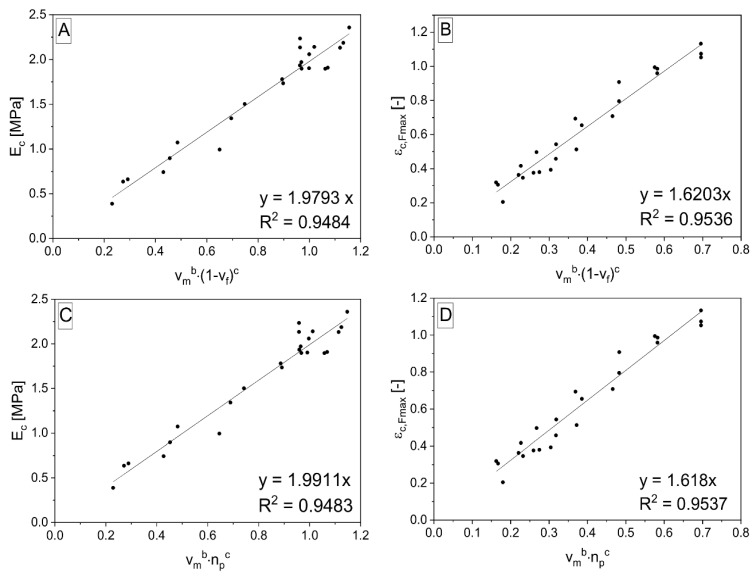
Linear fitting of plot between selected mechanical properties of porous composites composed of P_85_-G_5_-CO_10_ matrix filled by different fractions of rubber: *E* (**A**,**C**), *ε_Fmax_* (**B**,**D**); and expressions *v_m_^b^* (1 − *v_f_*)*^c^* (**A**,**B**) and *v_m_^b^*·*n_p_^c^* (**C**,**D**)—data fitted according to Equations (10) and (11).

There are three pairs of structural parameters and relevant spatial exponential functions, according to Table 3. One equation was published before [33], and the further suitable relationships are represented by Equations (10) and (11). The structural parameters are powered by exponents ensuring the highest value of R^2^ through the first fitting of linear function (the product of powered structural parameters is placed on the axis *x*). These parameters are labeled in the same way in both equations (also in Equation (4)), therefore, it is important to mention their different values in various equations. The fitting of measured data for the representative sample according to Equation (10) and (11) is shown in Figure 5.

(10) $$z_{c}=z_{m}\cdot v_{m}^{b}\cdot {\left(1-v_{f}\right)}^{c}$$

(11) $$z_{c}=z_{m}\cdot v_{m}^{b}\cdot n_{p}^{c}$$

where *z_c_* is the value of the chosen mechanical property (e.g., elastic modulus, ultimate strength, etc.) of composite material and *z_m_* is the value of the same property for nonporous matrix. The slope *z_m_* should correspond with the property value for hypothetical nonporous matrix (nonporous matrices were not available). The *z_m_* values obtained for different matrices by fitting to Equations (4) [33], (10), and (11) are shown in Table 4. The pair values valid for the combinations of matrix and property are similar. It is possible (with some extent of uncertainty) to compare fitted values of properties for any matrices according to Equations (10) and (11) in Table 4 with the real values for porous matrices in Table 2.

The porosity and density values of matrices in Table 2 could be also interesting. The maximal and minimal porosity for each combination matrix/filler are available in Table 5. The porosity increases with filler addition for slightly porous matrices, and the lowest porosity corresponds to the lowest filler content (20 wt.% if porosity is neglected). When the matrix contains a higher porosity rate, the porosity decreases to some filler content, and behind this minimum, it increases again. According to our conclusions, this phenomenon can be explained by the replacement of porous matrix by solid filler up to a certain point. The matrix is unable to fulfill the space among filler particles, and the porosity increases above this point. The highest porosity corresponds to the highest filler content for all composites. The pure matrices have the lowest porosity (Table 4) in comparison with that of composites with any filler content (Table 5). There is one exception—composites based on matrix designated P_99_-W_1_. This matrix is more porous than all prepared composites with the P_99_-W_1_ matrix.

When we compare the behavior of structural parameter pairs in Equations (4), (10), and (11), we can find some differences. The first pair *n_p_* and (1 − *v_f_*) (4) contains one parameter equal to one (*n_p_*), if we simplify the system to nonporous composite and 1 − *v_f_* in case of porous matrix. We get the same result for porous matrix (1 − *v_f_* equal to 1) but different for nonporous composite for the second parameter pair *v_m_* and 1 − *v_f_* in Equation (10). In this case, the value of both parameters is the same (*v_m_* = 1 − *v_f_*). The third pair, *n_p_* and *v_m_* from Equation (11), embodies the opposite behavior to the second (10). Interspace filling is equal to 1 in nonporous composite but for porous matrix *n_p_* = *v_m_*. The mentioned differences in structural pair behavior are connected with the behavior of *b* and *c* exponents in Equations (4), (10) and (11). The exponents show the interconnection of these three equations in some way. It is possible to write the equations where the exponents are replaced by numbers from fitting and then simplify the equations for nonporous composite or porous matrix. The behavior of exponents agrees with the behavior of structural parameters. The result is shown in the equations in Table 6.

**Figure 6:**
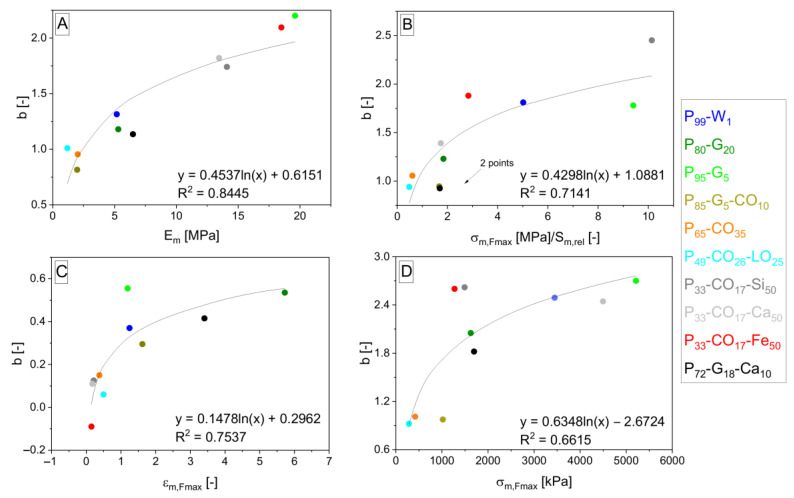
Logarithmic dependencies *y* = *d* + *e* · ln *x* of parameters *b* from general Equation (10) with different *b* values according to specification of property *z* into *E* (**A**), *σ_Fmax_* (**B**), *ε_Fmax_* (**C**) and *A_Fmax_* (**D**) interpolated by *E_m_* (**A**), *σ*_*m*,*Fmax*_/*S*_*m*,*rel*_ (**B**), *ε*_*m*,*Fmax*_ (**C**) and *σ*_*m*,*Fmax*_ (**D**) values from Table 4 except *S*_*m*,*rel*_ from Equation (20). The resulting dependencies correspond to exponent *d* + *e* · ln *x* in Equations (12)–(15) arising from Equation (10) in the order from (**A**–**D**).

**Figure 7:**
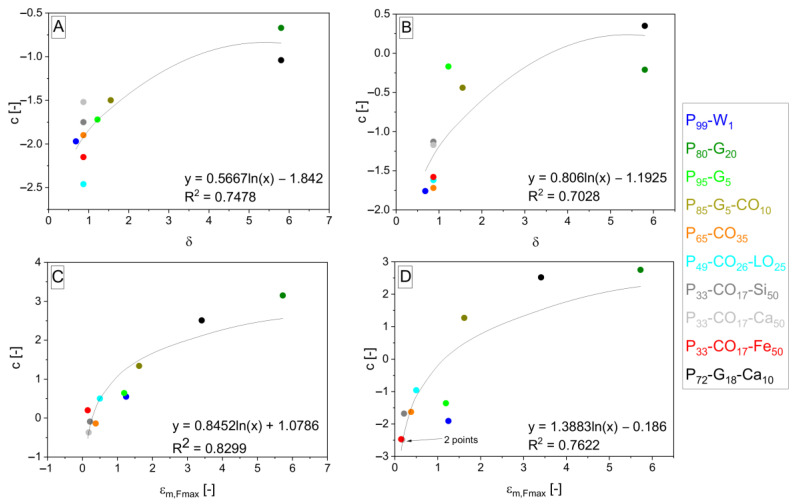
Logarithmic dependencies *y* = *f* + *g* · ln *x* of parameters *c* from general Equation (10) with different *c* values according to specification of property *z* into *E* (**A**), *σ_Fmax_* (**B**), *ε_Fmax_* (**C**), and *A_Fmax_* (**D**) interpolated by *δ* (**A**,**B**) and *ε*_*m*,*Fmax*_ (**C**,**D**) values from Table 1 (*δ*) and Table 4 (*ε*_*m*,*Fmax*_). The resulting dependencies correspond to exponent *f* + *g* · ln *x* in Equations (12)–(15) arising from Equation (10) in the order from (**A**–**D**).

**Figure 8:**
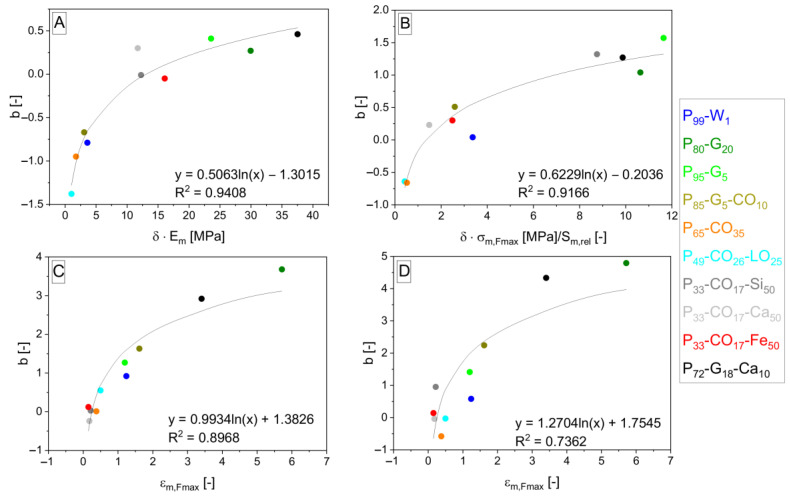
Logarithmic dependencies *y* = *d* + *e* · ln *x* of parameters *b* from general Equation (11) with different *b* values according to specification of property *z* into *E* (**A**), *σ_Fmax_* (**B**), *ε_Fmax_* (**C**), and *A_Fmax_* (**D**) interpolated by *δ*·*E_m_* (**A**), *δ*·*σ*_*m*,*Fmax*_/*S*_*m*,*rel*_ (**B**), and *ε*_*m*,*Fmax*_ (**C**,**D**) values from Table 1 (*δ*), Equation (20) (*S*_*m*,*rel*_), and Table 4 (the others). The resulting dependencies correspond to exponent *d* + *e* · ln *x* in Equations (16)–(19) arising from Equation (11) in the order from (**A**–**D**).

**Figure 9:**
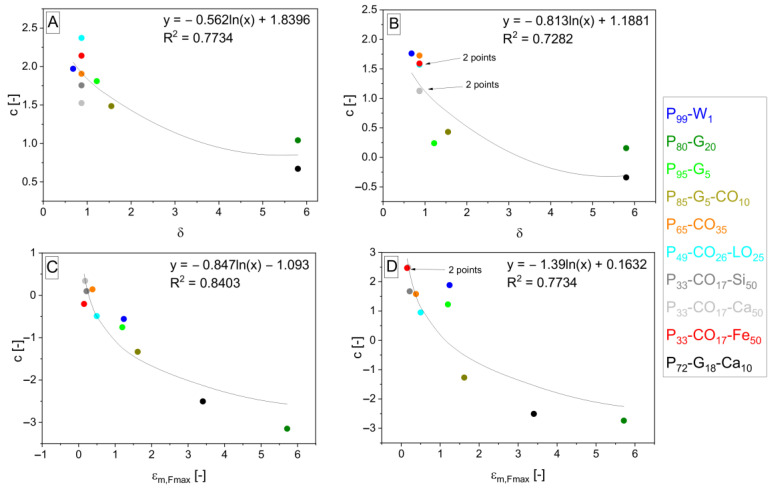
Logarithmic dependencies *y* = *f* + *g* · ln *x* of parameters *c* from general Equation (11) with different *c* values according to specification of property *z* into *E* (**A**), *σ_Fmax_* (**B**), *ε_Fmax_* (**C**), and *A_Fmax_* (**D**) interpolated by *δ* (**A**,**B**) and *ε*_*m*,*Fmax*_ (**C**,**D**) values from Table 1 (*δ*) and Table 4 (*ε*_*m*,*Fmax*_). The resulting dependencies correspond to exponent *f* + *g* · ln *x* in Equations (16)–(19) arising from Equation (11) in the order from (**A**–**D**).

The interpolation takes place and gives Equations (12)–(15) and (16)–(19) after the fitting, giving *b* and *c* parameters. Parameters *b* and *c* are interpolated by logarithmic functions (shown in Figure 6, Figure 7, Figure 8 and Figure 9). *S*_*m*,*rel*_ represents the relative integral area lying under the tensile curve of the (hypothetically) nonporous matrix in the Equations (13) and (17). It is explained by Equation (20). *S*_*m*,*rel*_ reaches the values in the range from 0 to 1 and was also applied [33] for interpolation of experimental data to create the relationship for *σ_c,Fmax_* calculation. The role of *δ* in the exponent functions (= polarity of matrix represented by OH/NCO ratio before curing) is getting important in the direction of Equations (7) > (12) > (16) valid for *E_c_* calculation. On the other hand, the role of slope (*E_m_*) in exponents is decreasing in the same direction. The same trend can be observed in relationships for *σ_Fmax_* calculation due to similar relationships for *E_c_* and *σ_c,Fmax_*.

Equations (12)–(19) have the same shape created by parameters with some differences according to described property and chosen structural parameters of materials. In this place, it is suitable to mention the attached overview tables and schemes (as figures) in the Supplementary Materials for a better explanation. Table S1 contains abbreviations explanations and Table S2 serves for a better explanation of the equation-creating method common for all properties represented by elastic modulus as example by explanation of mean and obtaining of all equation members through the whole process. The mathematical process leading from measured data to the obtained equations is depicted in Figure S3 in Supplementary Materials. The development of ideas during the research leading to obtained equation is shown as a flow chart depicted in figures in Supplementary Materials (Figure S4–S6).

(12) $$E_{c}=E_{m}\cdot v_{m}^{d+e\cdot \ln E_{m}}\cdot {\left(1-v_{f}\right)}^{f+g\cdot \ln\delta }$$

(13) $$\sigma_{c,F\max}=\sigma_{m,F\max}\cdot v_{m}{}^{d+e\cdot \ln\left(\frac{\sigma_{m,F\max}}{S_{m,rel}}\right)}\cdot {\left(1-v_{f}\right)}^{f+g\cdot \ln\delta }$$

(14) $$\varepsilon_{c,F\max}=\varepsilon_{m,F\max}\cdot v_{m}{}^{d+e\cdot \ln\varepsilon_{m,F\max}}\cdot {\left(1-v_{f}\right)}^{f+g\cdot \ln\varepsilon_{m,F\max}}$$

(15) $$A_{c,F\max}=A_{m,F\max}\cdot v_{m}^{d+e\cdot \ln\sigma_{m,F\max}}\cdot {\left(1-v_{f}\right)}^{f+g\cdot \ln\varepsilon_{m,F\max}}$$

(16) $$E_{c}=E_{m}\cdot v_{m}{}^{d+e\cdot \ln\left(E_{m}\cdot \delta \right)}\cdot n_{p}^{f+g\cdot \ln\delta }$$

(17) $$\sigma_{c,F\max}=\sigma_{m,F\max}\cdot v_{m}{}^{d+e\cdot \ln\left(\frac{\sigma_{m,F\max}\cdot \delta }{S_{m,rel}}\right)}\cdot n_{p}^{f+g\cdot \ln\delta }$$

(18) $$\varepsilon_{c,F\max}=\varepsilon_{m,F\max}\cdot v_{m}{}^{d+e\cdot \ln\varepsilon_{m,F\max}}\cdot n_{p}^{f+g\cdot \ln\varepsilon_{m,F\max}}$$

(19) $$A_{c,F\max}=A_{m,F\max}\cdot v_{m}{}^{d+e\cdot \ln\varepsilon_{m,F\max}}\cdot n_{p}^{f+g\cdot \ln\varepsilon_{m,F\max}}$$

(20) $$S_{m,rel}=\frac{A_{m,F\max}}{\sigma_{m,F\max}\cdot \varepsilon_{m,F\max}}=\sum \frac{\varepsilon_{n}-\varepsilon_{n-1}}{\varepsilon_{m,F\max}}\cdot \frac{\sigma_{n}+\sigma_{n-1}}{2\cdot \sigma_{m,F\max}};\;n\;\in \;{\langle 1;n_{F\max}\rangle }_{\;}$$

**Figure 10:**
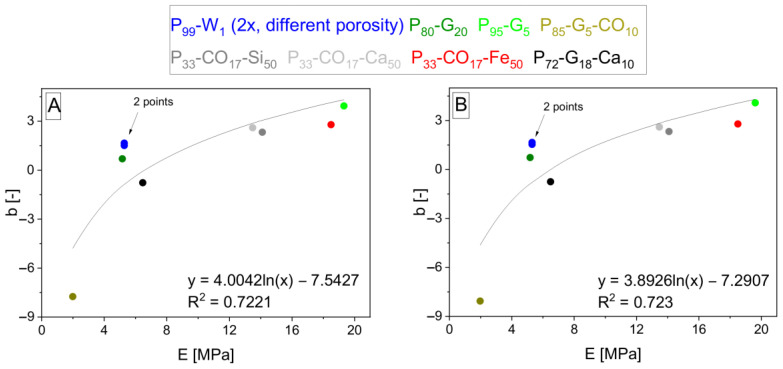
The logarithmic interpolation *y* = *d* + *e* · ln *x* of *b* parameters on *E_m_*. Parameters *b* were calculated by modified Equation (21) if the property *z* is specified as *E*, when *E_m_* values were taken from Table 4 according to Equations (10)–(11) (**A**,**B**) and the *E_mn_* and *n_m_* values were measured (see Table 2, used averages without deviations).

As mentioned above, proposed relationships can be simplified for the description of porous matrix. The form written in symbols is the same for simplification of both Equations (10) and (11). The reason is that, in this case, *v_m_* = *n_p_* (important for (11)), 1 − *v_f_* = 1 (important for (10)), and *v_m_* = 1 − *n_m_* (important for both of them). The general simplified relationship is Equation (21). The relevancy of this relationship can be proven very easily. The *z_mn_* (porous matrix) and *n_m_* can be measured (see Table 2). The *z_m_* values can be derived from the fitting of measured data valid for composite materials (see Table 4). The *z_m_* values are almost the same for (10) and (11), therefore, the calculated *b* values and then obtained dependencies are similar. The fitting results as examples for *E* are depicted in Figure 10. The shape of the resulting relationship is Equation (22).

(21) $$z_{mn}=z_{m}\cdot {\left(1-n_{m}\right)}^{b}$$

(22) $$E_{mn}=E_{m}\cdot {\left(1-n_{m}\right)}^{{}^{d+e\cdot \ln E_{m}}}$$

This article is an important supplement to the previous article [33]. It retains the earlier used method of data processing and uses the same primary data on one side. However, it introduces some new combinations of structural parameters and proves some of them (*n_p_* and *v_m_*, *v_m,_* and 1 − *v_f_*) as utilizable as the *n_p_* and 1 − *v_f_* combination [33]. These new combinations also ensure simplification of the equations to nonporous composite (theoretically) and porous matrix (tested) behavior description. The new proposed relationships are appropriate to the description of more complex composites containing two different fillers if one is included in the matrix. It is essential to mention here the wide scale of filling and porosity rates of materials included in this work.

This work enriches the possibilities of mathematical processing of the measured data and shows another way for further research in this field (the process is shown graphically in Figure S3). The possibility of connecting the exponents *b* and *c* values through proposed relationships is fascinating and can play a role in further research. The positive contribution of this work is the ability to find another way to describe the behavior of the totally random system on a microstructural level containing randomly shaped particles and voids.

The limits of this work are in the variability of fillers (chemically only rubber) because it was primarily focused on the utilization of different matrices. There were some particle size differences among the filler fractions, but approximately only in one mathematical order throughout all used rubber fillers. Moreover, there were quite wide distributions of separate fractions (in comparison with the total variability of rubber particles from all fractions). Therefore, the composition of samples did not enable suitable research in the field of different sizes of particles and other properties. Instead of researching influence of different particles on the equations connecting the behavior and composition of composites, it allowed neglecting filler fraction variability and using all composite samples in common calculations (fitting and interpolation). The essential role belonged to the choice of matrices. Matrices had a great advantage in high rates of natural porosity. However, this advantage was linked to a disadvantage of preparation inability of nonporous matrices.

Different kinds of filler with various shapes, distributions, and surface properties influencing the adhesion between filler and matrix should be included in the research in the future with the observation of relationship members. We expect a composition of similar dependencie(s) to those in this work of mechanical behavior on structure/composition when different particles (much more different than our rubbery) would be included with one matrix in one research. We expect the possibility of a combination of these dependencies or their connection into the one common for all types of matrices and fillers. Including naturally nonporous matrices in porous composites would be very interesting to enable direct comparison between fitting slopes and matrices properties values (the porosity would be ensured physically or chemically by the addition of extra chemicals or by the simple inability of matrix to fill space among filler particles in cases of too high filler contents). The ability of relationships to be fully utilizable will not be complete without the solving of mentioned ideas. These important insights (properties of filler and adhesion between filler and matrix depending on these components’ properties) should be included in research in the future.

## 4. Conclusions

This paper offers two new possibilities for how to link the structure and composition with studied mechanical properties (elastic modulus, ultimate strength and strain, energy need to ultimate strength achievement) of porous composite materials by proposed exponential relationships. Examined materials do not have any regular microstructure due to the utilization of randomly shaped particles as a filler, and the voids are also randomly shaped. These realities make the proposed equations interesting with research and practice potential in the future.

The relationships use special structural parameters called interspace volume, interspace filling, and volume fraction of matrix utilizable in pairs in the proposed equations. The article is dedicated to the introduction of relationships based on a combination of the matrix volume fraction with interspace filling or interspace volume.

The new relationships are created in two steps. The first step is the fitting of measured data, and the second step is the interpolation of parameters obtained by the fitting. The fitting step leads to the spatial exponential function with the slope, meaning the given property value valid for nonporous matrix. The second step (interpolation) leads to exponent description. This paper represents comprehensive research, but the relationships still do not have final forms and require deep research based on various parameters of fillers and their behavior with combinations of different matrices.

## Figures and Tables

**Table 1:** Chemical composition of PUR matrices (P—PU4223 CS; curing agents: W—water, G—glycerol, CO—castor oil; plasticizer: LO—linseed oil; fillers included in matrices: Si—SiO_2_, Ca—CaCO_3_, Fe—Fe). Table taken from previous work [33] *.

Designation	PU4223 CS (vol%)	Curing Agent (vol%)	Others (vol%)	*δ* ^a^ (-)	DBTL ^b^ (wt%)
P_99_-W_1_	99	1 (W)	-	0.68 ^c^	0.1
P_95_-G_5_	95	5 (G)	-	1.22	0.03
P_80_-G_20_	80	20 (G)	-	5.79	0.03
P_85_-G_5_-CO_10_	85	5 (G) + 10 (CO)	-	1.55	0.03
P_65_-CO_35_	65	35 (CO)	-	0.87	0.1
P_49_-CO_26_-LO_25_	49	26 (CO)	25 (LO)	0.87	0.1
P_33_-CO_17_-Si_50_	33	17 (CO)	50 (Si)	0.87	0.1
P_33_-CO_17_-Ca_50_	33	17 (CO)	50 (Ca)	0.87	0.1
P_33_-CO_17_-Fe_50_	33	17 (CO)	50 (Fe)	0.87	0.1
P_72_-G_18_-Ca_10_	72	18 (G)	10 (Ca)	5.79	0.03

^a^ Reflecting OH/NCO molar ratio; ^b^ wt% of Di-butyl-tin-di-laureate. Based on the weight of PU 4223 CS; ^c^ For this purpose, H_2_O represents two OH groups. * Reprinted by permission from Springer Nature Customer Service Centre GmbH: Springer Nature, SN Applied Sciences, A new approach to the structure-properties relationship evaluation for porous polymer composites, Cerny et al., 2020, https://doi.org/10.1007/s42452-020-2479-8, accessed on 22 March 2023 [SN Appl. Sci.], https://www.springer.com/journal/42452, accessed on 22 March 2023.

**Table 2:** Mechanical properties (elastic modulus *E*, ultimate strength *σ_Fmax_*, ultimate strain *ε_Fmax,_* and energy need corresponding to ultimate strength achievement *A_Fmax_*), density of theoretical nonporous matrix *ρ_t,_* and whole porosity *n_m_* of PUR matrices. Table taken from previous work [33] *.

Designation	*E* (MPa)	*σ_Fmax_* (MPa)	*ε_Fmax_* (%)	*A_Fmax_* (kJ·m^−3^)	*ρ_t_* (g·cm^−3^)	*n_m_* (%)
P_99_-W_1_	1.4 ± 0.3	0.56 ± 0.05	94 ± 9	321 ± 14	1.10 ^a^	57 ± 7
P_99_-W_1_	4.1 ± 0.1	2.7 ± 0.3	82 ± 12	1400 ± 400	1.10 ^a^	16 ± 2
P_95_-G_5_	13 ± 2	4.7 ± 0.1	140 ± 40	4100 ± 700	1.12 ^b^	9.5 ± 0.7
P_80_-G_20_	4.8 ± 0.3	1.75 ± 0.08	340 ± 20	3700 ± 300	1.15 ^b^	10 ± 1
P_85_-G_5_-CO_10_	2.3 ± 0.3	1.24 ± 0.04	100 ± 4	700 ± 50	1.05 ^b^	2 ± 2
P_65_-CO_35_	1.6 ± 0.4	0.42 ± 0.03	39 ± 4	69 ± 6	1.04 ^b^	4 ± 1
P_49_-CO_26_-LO_25_	1.1 ± 0.03	0.4 ± 0.03	68 ± 8	90 ± 8	1.03 ^b^	1 ± 2
P_33_-CO_17_-Si_50_	9 ± 1	2.2 ± 0.3	22 ± 1	210 ± 40	1.85 ^c^	16 ± 1
P_33_-CO_17_-Ca_50_	4.9 ± 0.5	0.7 ± 0.04	20 ± 1	69 ± 9	1.86 ^c^	32 ± 2
P_33_-CO_17_-Fe_50_	9.1 ± 0.6	1.09 ± 0.09	13 ± 1	70 ± 10	4.04 ^c^	22.6 ± 0.5
P_72_-G_18_-Ca_10_	7.4 ± 0.8	1.93 ± 0.06	260 ± 30	3700 ± 300	1.30 ^c^	16.3 ± 0.6

^a^ Measured by a pycnometer method; ^b^ Obtained from knowledge about volume, mass, and porosity; ^c^ Calculated via simple mixing equation. * Reprinted by permission from Springer Nature Customer Service Centre GmbH: Springer Nature, SN Applied Sciences, A new approach to the structure-properties relationship evaluation for porous polymer composites, Cerny et al., 2020, https://doi.org/10.1007/s42452-020-2479-8, accessed on 22 March 2023, [SN Appl. Sci.], https://www.springer.com/journal/42452, accessed on 22 March 2023.

**Table 3:** Suitability of all possible 10 structural parameter pairs for research. Unsuitable pairs were excluded immediately after finding some insufficiency without searching for their complex behavior.

Parameter	Suitability or Reason for Exclusion
1 − *n*/*n_p_*	– nonporous composite: (1 − *n*) · *n_p_* = 1
*n_pf_*/1 − *v_f_*	– porous matrix: *n_pf_* · (1 − *v_f_*) = 1
1 − *n*/1 − *v_f_*; *n_pf_*/*v_m_*; *n_pf_*/1 − *n*	– first fitting: Low R^2^ in comparison with the fitting according to pair *n_p_*/1 *– vf* [33]
1 − *n*/*v_m_*; *n_pf_*/*n_p_*	– too different slope value in the first fitting in comparison with Table 4
*n_p_*/1 − *v_f_* [33]; *v_m_*/1 − *v_f_*; *v_m_*/*n_p_*	– suitable for further research

**Table 4:** Mechanical properties of theoretically nonporous matrices obtained by fitting of composite mechanical properties according to Equations (4) [33], (10) and (11).

Matrix	*E* (MPa)	*σ_Fmax_* (MPa)	*ε_Fmax_* (-)	*A_Fmax_* (kJ·m^−3^)
P_99_-W_1_	5.30/5.31/5.28	3.38/3.45/3.39	1.25/1.25/1.24	2871/2961/2879
P_95_-G_5_	19.38/19.62/19.32	5.17/5.21/5.21	1.19/1.19/1.20	3377/3448/3416
P_80_-G_20_	5.17 5.18/5.17	1.49/1.49/1.49	5.72/5.73/5.72	6920/6923/6911
P_85_-G_5_-CO_10_	1.98/1.98/1.99	1.02/1.02/1.02	1.62/1.62/1.62	1004/1004/1002
P_65_-CO_35_	2.02/2.03/2.02	0.42/0.42/0.42	0.38/0.38/0.38	111/111/113
P_49_-CO_26_-LO_25_	1.19/1.19/1.20	0.29/0.28/0.29	0.50/0.50/0.50	83/83/83
P_33_-CO_17_-Si_50_	14.09/14.09/14.11	4.50/4.50/4.49	0.22/0.22/0.22	435/434/435
P_33_-CO_17_-Ca_50_	13.46/13.47/13.48	1.28/1.28/1.27	0.19/0.19/0.18	167/167/168
P_33_-CO_17_-Fe_50_	18.53/18.51/18.50	1.70/1.70/1.70	0.14/0.15/0.15	153/152/153
P_72_-G_18_-Ca_10_	6.48/6.49/6.47	1.62/1.63/1.63	3.41/3.41/3.41	5320/5309/5299

**Table 5:** Maximal and minimal values of porosity (*n_max_*, *n_min_*) in composites for all combinations of matrices and rubbery fillers with mentioned corresponding volume fractions of filler (*v*_*f*(*t*)_—theoretical value meaning neglecting of porosity). Porosity values are averages from measurement of five samples.

PUR Matrix	*n_max_* (*v_f(t)_*)/*n_min_* (*v*_*f*(*t*)_)—(%) (%)/(%) (%), If Filler Is:		
	R_2_	R_1_	R_0_
P_99_-W_1_	45 (90)/32 (70)	53 (90)/36 (70)	54 (90)/28 (50)
P_95_-G_5_	46 (90)/15 (20)	54 (90)/26 (20)	45 (90)/24 (20)
P_80_-G_20_	46 (90)/12 (20)	44 (90)/15 (40)	46 (80)/14 (30)
P_85_-G_5_-CO_10_	44 (90)/7 (20)	42 (90)/12 (20)	48 (90)/12 (20)
P_65_-CO_35_	46 (90)/10 (30)	50 (90)/12 (20)	50 (90)/15 (20)
P_49_-CO_26_-LO_25_	47 (90)/6 (20)	49 (90)/4 (20)	50 (90)/8 (20)
P_33_-CO_17_-Si_50_	42 (80)/13 (40)	45 (80)/13 (40)	43 (70)/12 (30)
P_33_-CO_17_-Ca_50_	47 (90)/16 (50)	44 (80)/15 (40)	47 (80)/18 (30)
P_33_-CO_17_-Fe_50_	44 (80)/16 (40)	45 (80)/17 (40)	47 (80)/15 (30)
P_72_-G_18_-Ca_10_	44 (90)/13 (50)	51 (90)/15 (40)	45 (80)/14 (40)

**Table 6:** Mathematical behavior of structural parameter pairs and their exponents coming from Equations (4), (10) and (11). The behavior is shown by the example of data fitting connecting the composites based on P_95_-G_5_ matrix and their values of ultimate strength. The behavior of exponents could also be supplemented by Equation *c*(11) =−*b*(10) = *b* − *c*(4), that is not in the table due to a missing connection to equation simplification if the material is also simplified.

Powered Parameters	Mathematical Treatment	Exponents Behavior (eq.)	Notice
hypothetical nonporous composite			
$n_{p}^{1.78}\cdot {\left(1-v_{f}\right)}^{1.60}$	${\left(1-v_{f}\right)}^{1.60}$	c(4)=b+c(10)=b(11)	$n_{p}=1$
$v_{m}^{1.78}\cdot {\left(1-v_{f}\right)}^{-0.17}$	${\left(1-v_{f}\right)}^{1.61}$	b+c(10)=c(4)=b(11)	$v_{m}=1-v_{f}$
$v_{m}{}^{1.57}\cdot n_{p}^{0.24}$	${\left(1-v_{f}\right)}^{1.57}$	b(11)=c(4)=b+c(10)	$n_{p}=1;\;\;v_{m}=1-v_{f}$
porous matrix			
$n_{p}^{1.78}\cdot {\left(1-v_{f}\right)}^{1.60}$	$v_{m}{}^{1.78}$	b(4)=b(10)=b+c(11)	$1-v_{f}=1;\;\;n_{p}=v_{m}$
$v_{m}^{1.78}\cdot {\left(1-v_{f}\right)}^{-0.17}$	$v_{m}{}^{1.78}$	b(10)=b(4)=b+c(11)	$1-v_{f}=1$
$v_{m}{}^{1.57}\cdot n_{p}^{0.24}$	$v_{m}{}^{1.81}$	b+c(11)=b(4)=b(10)	$n_{p}=v_{m}$
porous composite (full equation shape, the example of fitting result)			
$\sigma_{c,F\max}\;\left(\mathrm{MPa}\right)=5.168\;\mathrm{MPa}\cdot n_{p}^{1.78}\cdot {\left(1-v_{f}\right)}^{1.60}$			
$\sigma_{c}{}_{,F\max}\;\left(\mathrm{MPa}\right)=5.212\;\mathrm{MPa}\cdot v_{m}^{1.78}\cdot {\left(1-v_{f}\right)}^{-0.17}$			
$\;\sigma_{c}{}_{,F\max}\;\left(\mathrm{MPa}\right)=5.215\;\mathrm{MPa}\cdot v_{m}{}^{1.57}\cdot n_{p}^{0.24}$			

## Data Availability

The data presented in this study are available on request from the corresponding author.

## Supplementary Materials

The following supporting information can be downloaded at: https://www.mdpi.com/article/10.3390/polym15081960/s1, Table S1: List of abbreviations and symbols in this work (except physical units); Table S2: Member meaning and mean of their obtaining for totally all equations contributing to elastic modulus calculation from the general beginning (getting structural parameters) to the end (porous matrix including). The other properties are not mentioned because of the same calculation method; Figure S1: Polyurethane chemistry related to curing of matrices. The scheme contains the used components formulas [39,40] (approximate in case of pre-polymer containing 10–30 wt% MDI—methylene-di-phenyl-di-isocyanate) and basic chemical reactions leading to polyurethanes (polyureas); Figure S2: The schematic overview of preparation and testing of samples and following data and style of their utilization in further mathematical processing; Figure S3: Data obtaining and processing—from beams to final equations; Figure S4: Flow chart including the thinking process through the whole research—1st part; Figure S5: Flow chart including the thinking process through the whole research—2nd part; Figure S6: Flow chart including the thinking process through the whole research—3rd part. It is necessary to add that Equation (3) (valid for 1-component materials in the form telling that exponents are number, not function) is mathematically at the beginning, but mentally is in the end. The results occurring in the literature belonging to 1-component materials were found after the research to add a suitable background. The results not including models/simulations for porous composites were looked for at the beginning of the research without success.

## References

[1] Choren J.A., Heinrich S.M., Silver-Thorn M.B. (2013). Young’s modulus and volume porosity relationships for additive manufacturing applications. J. Mater. Sci..

[2] Krishna V., Bose S., Bandyopadhyay A. (2007). Low stiffness porous Ti structures for load-bearing implants. Acta Biomater..

[3] Rubshtein A.P., Trakhtenberg I.S., Makarova E.B., Triphonova E.B., Bliznets D.G., Yakovenkova L.I., Vladimirov A.B. (2014). Porous material based on spongy titanium granules: Structure, mechanical properties, and osseointegration. Mat. Sci. Eng. C-Mater..

[4] Kovacik J. (2001). Correlation between shear modulus and porosity in porous materials. J. Mater. Sci. Lett..

[5] Lian C., Zhuge Y., Beecham S. (2011). The relationship between porosity and strength for porous concrete. Constr. Build. Mater..

[6] Fan X., Case E.D., Ren F., Shu Y., Baumann M.J. (2012). Part II: Fracture strength and elastic modulus as a function of porosity for hydroxyapatite and other brittle materials. J. Mech. Behav. Biomed..

[7] Karthikeyan S., Balasubramanian V., Rajendran R. (2014). Developing empirical relationships to estimate porosity and Young’s modulus of plasma sprayed YSZ coatings. Appl. Surf. Sci..

[8] Kovacik J. (1999). Correlation between Young’s modulus and porosity in porous materials. J. Mater. Sci. Lett..

[9] Kovacik J. (2008). Correlation between elastic modulus, shear modulus, Poisson’s ratio and porosity in porous materials. Adv. Eng. Mater..

[10] Smolin L.Y., Eremin M.O., Makarov M.P., Evtushenko E.P., Kulkov S.N., Buyakova S.P. (2014). Brittle Porous Material Mesovolume Structure Models and Simulation of their Mechanical Properties. AIP Conf. Proc..

[11] Werner J., Aneziris C.G., Schaffoner S. (2014). Influence of porosity on Young’s modulus of carbon-bonded alumina from room temperature up to 1450 °C. Ceram. Int..

[12] Zhang L., Gao K.W., Elias A., Dong Z.G., Chen W.X. (2014). Porosity dependence of elastic modulus of porous Cr3C2 ceramics. Ceram. Int..

[13] Sapozhnikov S.B., Kudryavtsev O.A., Dolganina N.Y. (2015). Experimental and numerical estimation of strength and fragmentation of different porosity alumina ceramics. Mater. Design.

[14] Wu Z., Sun L.C., Wang J.Y. (2016). Synthesis and characterization of porous Y2SiO5 with low linear shrinkage, high porosity and high strength. Ceram. Int..

[15] Sonnenschein M.F. (2003). Porosity-Dependent Young’s Modulus of Membranes from Polyetherether Ketone. J. Polym. Sci. Pol. Phys..

[16] Palchik V. (1999). Influence of porosity and elastic modulus on uniaxial compressive strength in soft brittle porous sandstones. Rock Mech. Rock Eng..

[17] Gibson L.J., Ashby M.F., Easterling K.E. (1988). Structure and mechanics of the iris leaf. J. Mater. Sci..

[18] Nielsen L.F. (1984). Elasticity and damping of porous materials and impregnated materials. J. Am. Ceram. Soc..

[19] Huiru X., Quizhen S. (2018). Deformation mechanisms and mechanical properties of porous magnesium/carbon nanofiber composites with different porosities. J. Mater. Sci..

[20] Wagh A.S., Poeppel R.B., Singh J.P. (1991). Open pore description of mechanical properties of ceramics. J. Mater. Sci..

[21] Chen Y., Xu Y.F. (2019). Compressive Strength of Fractal-Textured Foamed Concrete. Fractals.

[22] Bruck H.A., Rabin B.H. (1999). Evaluating microstructural and damage effects in rule-of-mixtures predictions of the mechanical properties of Ni-Al2O3 composites. J. Mater. Sci..

[23] Chao X.J., Tian W.L., Xu F., Shou D.H. (2021). A fractal model of effective mechanical properties of porous composites. Compos. Sci. Technol..

[24] Katsube N., Wu Y.N. (1998). A constitutive theory for porous composite materials. Int. J. Solids Struct..

[25] Xu H., Li Q. (2017). Effect of carbon nanofiber concentration on mechanical properties of porous magnesium composites: Experimental and theoretical analysis. Mater. Sci. Eng. A.

[26] Odhiambo J.O., Yoshida M., Otsu A., Yi L.-F., Onda T., Chen Z.-C. (2022). Microstructure and tensile properties of in-situ synthesized and hot-extruded aluminum-matrix composites reinforced with hybrid submicron-sized ceramic particles. J. Compos. Mater..

[27] Poh L., Della C., Ying S., Goh C., Li Y. (2015). Micromechanics model for predicting effective elastic moduli of porous ceramic matrices with randomly oriented carbon nanotube reinforcements. AIP Adv..

[28] Alam P. (2010). A mixtures model for porous particle-polymer composites. Mech. Res. Commun..

[29] Choi H.K., Son M.J., Shin E.S., Yu J. (2018). Prediction of thermos-poro-elastic properties of porous composites using an expanded unmixing-mixing model. Compos. Struct..

[30] Chan C., Naguib H.E. (2010). Development and Characterization of Polypyrrole-Polylactide Conductive Open-Porous Composites. J. Appl. Polym. Sci..

[31] Tran A.T., Le Quang H., He Q.-C. (2016). Computation of the size-dependent elastic moduli of nano-fibrous and nano-porous composites by FFT. Compos. Sci. Technol..

[32] Khoroshun L.P., Shikula E.N. (1993). Nonlinear Straining of Porous Composite Materials. Int. Appl. Mech..

[33] Cerny M., Petrus J., Kucera F., Pavlinakova V., Kupka V., Polacek P., Chamradova I. (2020). A new approach to the structure-properties relationship evaluation for porous polymer composites. SN Appl. Sci..

[34] Keleş Ö., Anderson E.H., Huynh J., Gelb J., Freund J., Karakoç A. (2018). Stochastic fracture of additively manufactured porous composites. Sci. Rep..

[35] Teng J., Yang B., Zhang L.-Q., Lin S.-Q., Xu L., Zhong G.-J., Tang J.-H., Li Z.-M. (2018). Ultra-high mechanical properties of porous composites based on regenerated cellulose and cross-linked poly(ethylene glycole). Carbohydr. Polym..

[36] Węgrzyk S., Herman D. (2021). Strengthening of Al_2_O_3_ porous composites with a glass-ceramic binder doped with nanocopper. J. Eur. Ceram..

[37] Olmos L., Gonzalés-Pedraza A.S., Vergara-Hernández H.J., Chávez J., Jimenez O., Mihalcea E., Arteaga D., Ruiz-Mondragón J.J. (2022). Ti64/20Ag Porous Composites Fabricated by Powder Metallurgy for Biomedical Applications. Materials.

[38] Seuba J., Maire E., Adrien J., Meille S., Deville S. (2021). Mechanical properties of unidirectional porous polymer/ceramic composites for biomedical applications. Open Ceram..

[39] Yeboah A., Ying S., Lu J., Xie Y., Amoanimaa-Dede H., Boateng K.G.A., Chen M., Yin X. (2021). Castor oil (Ricinus communis): A review on the chemical composition and physicochemical properties. Food. Sci. Technol..

[40] Vereshchagin A.G., Novitskaya G.V. (1965). The triglyceride composition of linseed oil. J. Am. Oil Chem. Soc..

