# Supplementary material for: The Influence of Porosity on Mechanical Properties of PUR-Based Composites: Experimentally Derived Mathematical Approach
Source: Polymers (Basel). 2023 Apr 20;15(8):1960. doi: 10.3390/polym15081960 (PMC10141045; doi:10.3390/polym15081960)
Supplement: Supplementary file 1 [file polymers-15-01960-s001.zip › polymers-2332152-supplementary.pdf]

Supplementary materials

Table S1. List of abbreviations and symbols in this work (except physical units).

**Properties**	
z, z_c, z_{mn}, z_m	– property without specification—generally, for composite materials, porous and non-porous matrix
E, E_m, E_{mn}, E_c	– elastic modulus—generally, for non-porous matrix, porous matrix, composite
σ_{Fmax}, $\sigma_{m,Fmax}$, $\sigma_{c,Fmax}$	– ultimate strength—generally, for non-porous matrix, composite
ε_{Fmax}, $\varepsilon_{m,Fmax}$, $\varepsilon_{c,Fmax}$	– ultimate strain—generally, for non-porous matrix, composite
A_{Fmax}, $A_{m,Fmax}$, $A_{c,Fmax}$	– energy need for ultimate strength achievement—generally, for non-porous matrix, composite
$S_{m,rel}$	– relative area lying below tensile curve up to ultimate strength achievement in the case of non-porous matrix (obtained by calculation, measured is not available)
**Structural parameters**	
n, n_m	– porosity (generally), matrix porosity
n_{max}, n_{min}	– maximal and minimal porosity
ρ, $\rho_{t,}$, ρ_{it}	– density (generally), theoretical density of sample (porosity neglecting) resp. of its component
v_{it}	– volume fraction of ith component in material
v_m, $v_{m(t)}$	– matrix volume fraction—real and with porosity neglecting
n_p	– interspace filling
v_f, $v_{f(t)}$	– volume fractions of filler—real and with porosity neglecting
$1-v_f$	– interspace volume
$1-n$	– solid rate of material
n_{pf}	– matrix rate in solid rate of material = matrix volume fraction if the porosity is neglected
m, *wt.%*, *V*, *vol.%*	– mass, weight percent, volume, volume percent
**Remaining parameters**	
b, *c*	– exponents ensuring the best fitting of spatial exponential function serving for filled porous composites mechanical behavior description (different properties = different *b, c* values)
d, *e*, *f*, *g*	– parameters arising through *b* and *c* fitting by matrix properties
δ	– OH/NCO rate in polyurethane matrix before curing, substituting the matrix polarity and adhesion of matrix for other material components
**Chemical components designations**	
P	– pre-polymer Unixin PU4223 based on MDI
MDI	– methylene-di-phenyl-di-isocyanate
W, G, CO	– curing agents—water, glycerol, castor oil
LO, Si, Ca, Fe	– matrix modifiers—linseed oil, quartz, calcite, iron
R_0, R_1, R_2	– rubbery filler fractions
DBTL	– di-butyl-tin-di-laureate

Table S2. Member meaning and mean of their obtaining for totally all equations contributing to elastic modulus calculation from the general beginning (getting structural parameters) to the end (porous matrix including). The other properties are not mentioned because of the same calculation method.

**Parameter**	**Significance in Equations (1), (5), (6), (8-9)**	**Derived from…**
n_p	interspace filling	calculated by Equation (5)
n	sample porosity	calculated by Equation (1)
$v_{m(t)}$	matrix volume fraction in hypothetical nonporous material	known (adjusted)
$1\text{-}v_f$	interspace volume	calculated by Equation (6)
$1\text{-}n$	solid rate of material	calculated from porosity (by Equation (1))
v_m	volume fraction of matrix	calculated by Equation (8)
n_{pf}	matrix rate in solid rate of material	calculated by Equation (9)
**Parameter**	**Significance in Equations (10), (12)**	**Derived from…**
E_c	E value of composite	measured (10,12)
E_m	E value for nonporous matrix	obtained by fitting in (10) and then used in (12)
v_m	volume fraction of matrix	calculated from porosity (by Equation (1)) and composition (known)
$1\text{-}v_f$	interspace volume	calculated by Equation (6)
b, c (10)	exponents	values ensuring the highest R^2
d, e (12)	b exponent parameters	fitting of b according to E_m in (12)
f, g (12)	c exponent parameters	fitting of c according to δ (12)
δ (12)	OH/NCO ratio in uncured matrix	known (adjusted)
**Parameter**	**Significance in Equations (11), (15)**	**Derived from…**
n_p*	interspace filling	calculated by Equation (5)
d, e (15)	b exponent parameters	b fitting according to E_m and δ (16)
the other members	same meaning as in Equations (10,12)	corresponding to Equations (10,12)
**Parameter**	**Significance in Equations (21), (22)**	**Derived from…**
E_{mn}	E value for porous matrix	measured
E_m	E value for nonporous matrix	from Equations (10) or (11)
n_m	porosity of porous matrix	measured
b	exponent	calculated by Equation (21)
d, e (22)	b exponent parameters	b fitting according to E_m in (22)

* instead of $1\text{-}v_f$ in (10,12)

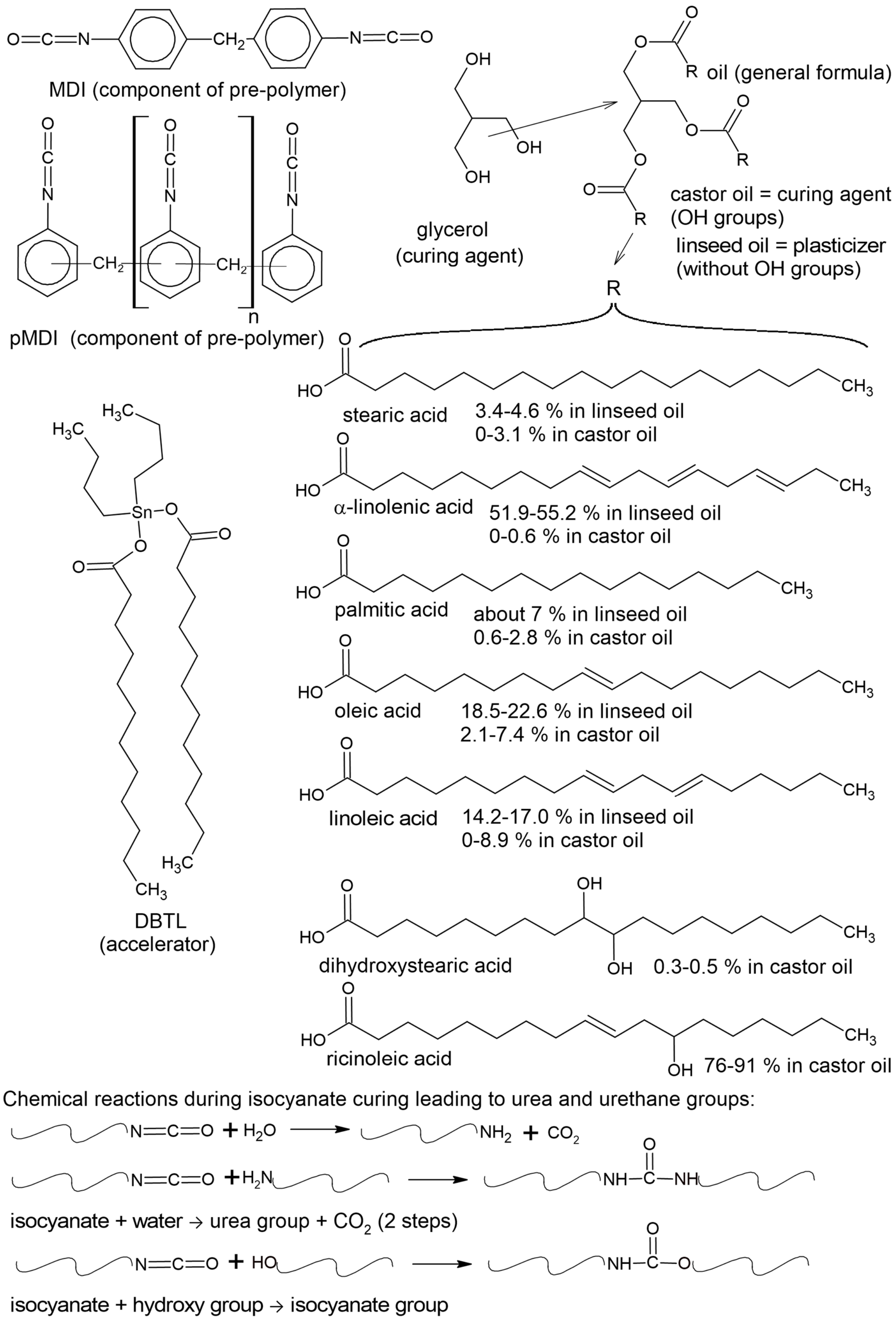

Figure S1. Polyurethane chemistry related to curing of matrices. The scheme contains the used components formulas [39,40] (approximate in case of pre-polymer containing 10-30 wt% MDI—methylene-di-phenyl-di-isocyanate) and basic chemical reactions leading to polyurethanes (polyureas).

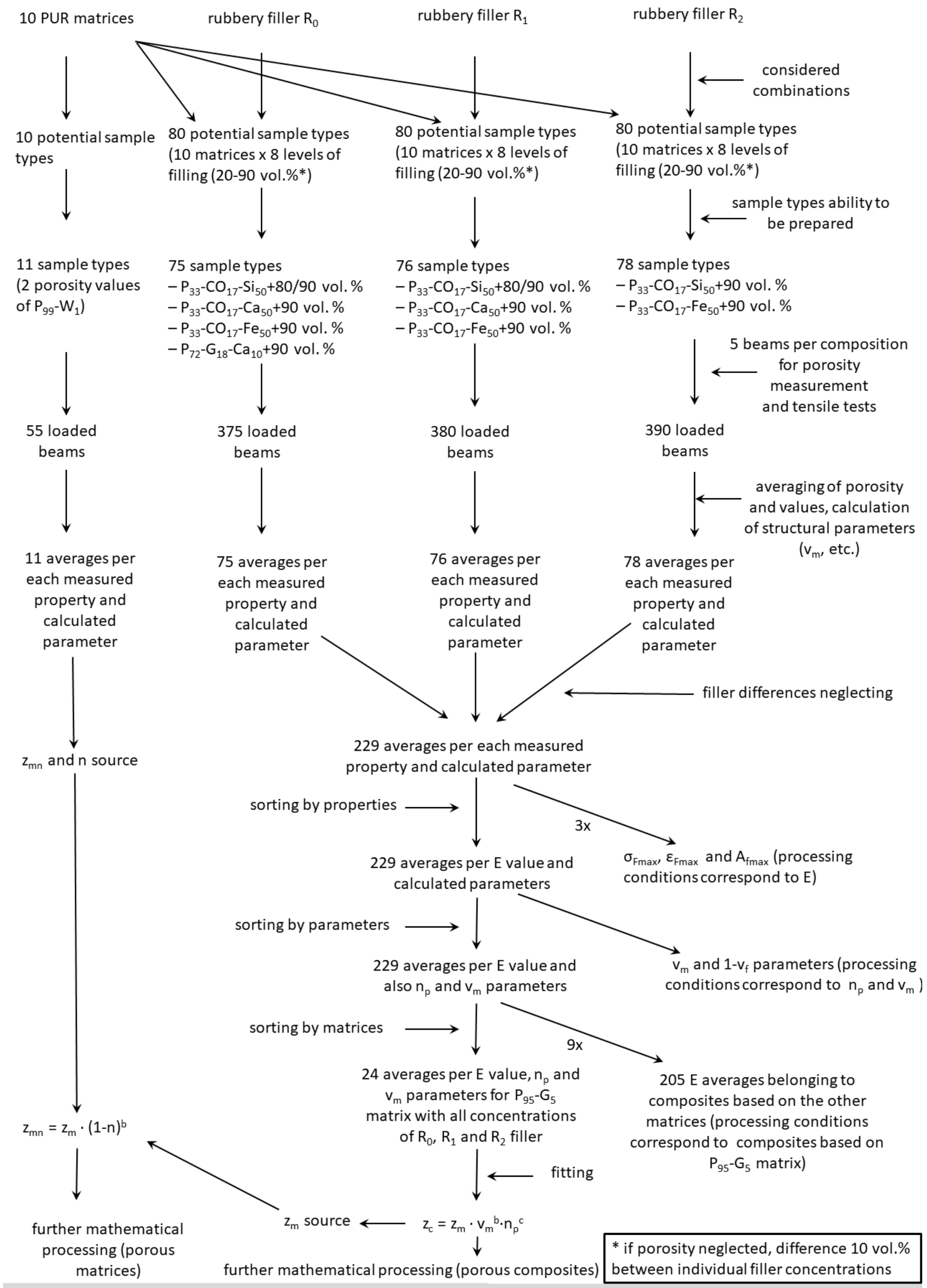

List of symbols:

z	– label of general property	A_{Fmax}	– energy need for ultimate strength achievement
z_m/z_{mn}	– general property value for nonporous/porous matrix	n_p	– interspace filling
z_c	– general property value for composite	1-v_f	– interspace volume
E	– tensile modulus	v_m	– matrix volume fraction
σ_{Fmax}	– ultimate strength	n	– porosity
ε_{Fmax}	– ultimate strain	b, c	– members depending on matrix properties
		others	– matrices designations are placed in Table 1

Figure S2. The schematic overview of preparation and testing of samples and following data and style of their utilization in further mathematical processing.

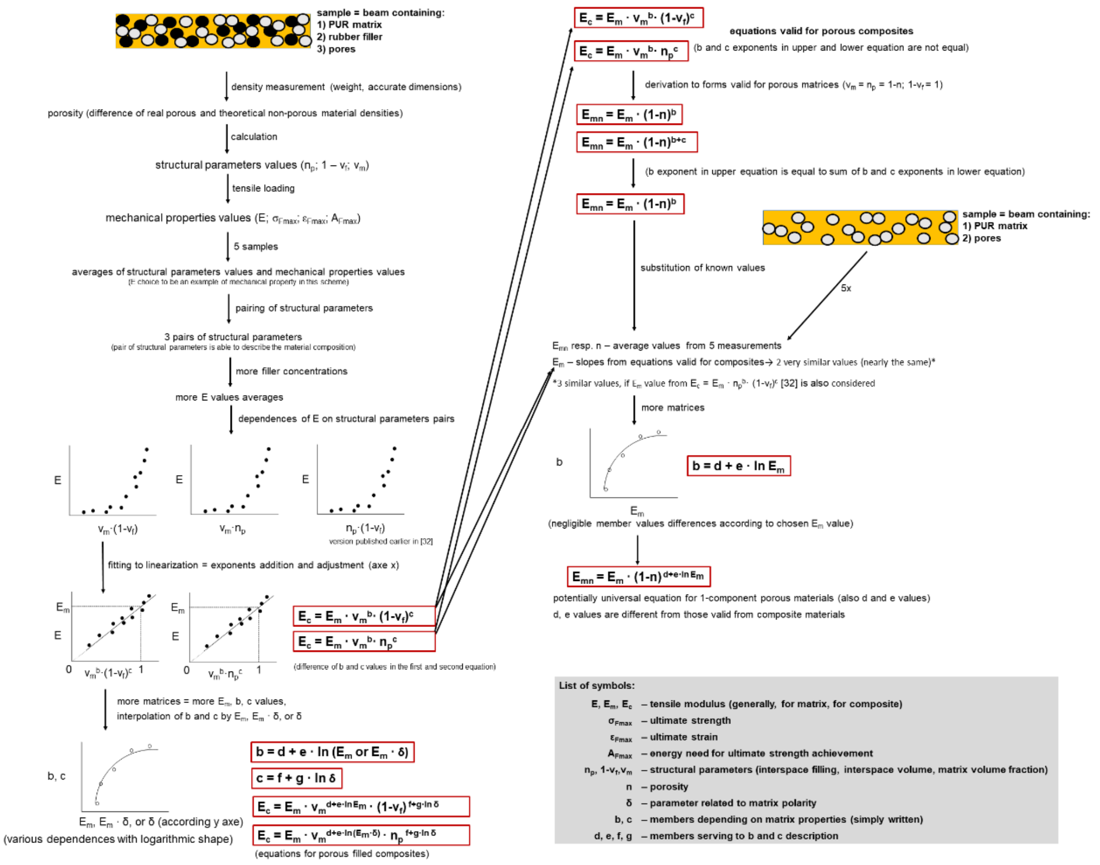

Figure S3. Data obtaining and processing—from beams to final equations.

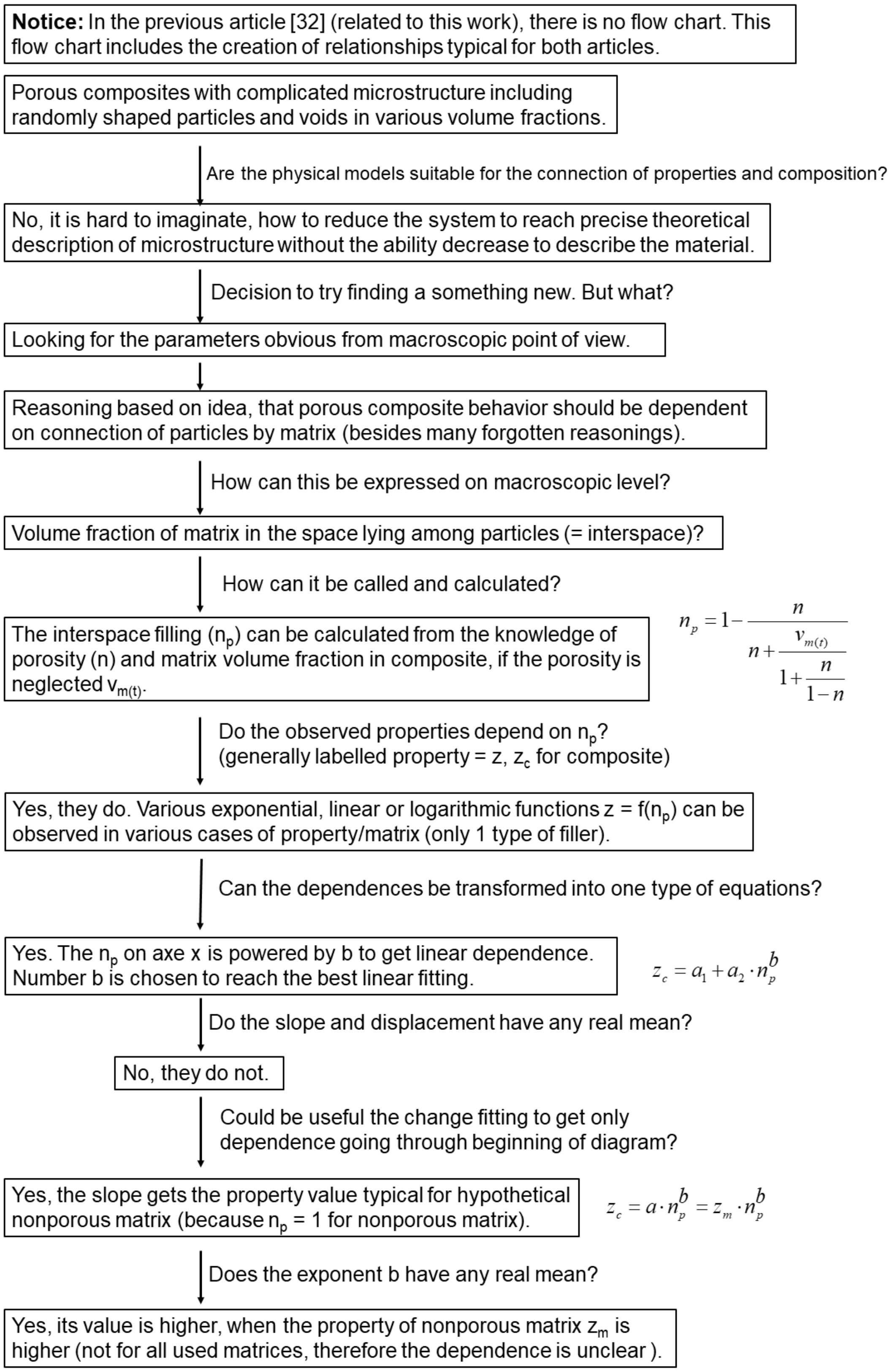

Figure S4. Flow chart including the thinking process through the whole research—1st part.

The obtained result seems interesting but incomplete. $z_c = a \cdot n_p^b = z_m \cdot n_p^b$

↓ Where is the mistake?

Awareness, that n_p is equal to 1 not only to nonporous matrix but also for nonporous composite) and this can do problems. The equation needs some member(s) more.

↓ Could the further structural parameter be useful?

Yes, it could, because there is necessity to differ nonporous matrix and nonporous composite. Attempts to employ composite density, porosity …

↓

New attempt was not fully succesful (b description, extent of fitting succes).

$z_c = z_m \cdot (n_p \cdot p_2)^b; \ldots$

↓ Could the further exponent (c) be useful?

Yes, it could (better fitting), but it has to power the second structural parameter.

↓ Is this result sufficient?

$z_c = z_m \cdot n_p^b \cdot p_2^c$

The fitting succes is different accroding to chosen parameter. The winner is $1\text{-}v_f$ (interspace filling, v_f is volume fraction of filler).

$$1 - v_f = n \cdot \left(1 + \frac{n_p}{1 - n_p}\right)$$

$$z_c = z_m \cdot n_p^b \cdot (1 - v_f)^c \quad [32]$$

↓ Is the choice of the second parameter according to fitting success useful for exponents decription?

Partially. There are logarithmic dependences, but not for all properties.

$$b = d + e \cdot \ln z_m$$
$$c = f + g \cdot \ln z_m$$

↓ Is it possible to make the explanation (interpolation) of the b and c exponents more accurately?

$$c = f + g \cdot \ln(z_m \cdot \delta)$$

Yes, it is after addition of expected polarity (δ) of matrix (or another properties) into logaritmic part of b or c dependences in certain cases.

↓ The obtained equation seems to be good for porous composites. Can its shape reflect the less complexity of material?

$$z_{mn} = z_m \cdot n_p^b \quad [32]$$
$$z_c = z_m \cdot (1 - v_f)^c \quad [32]$$

Yes, because n_p is equal to 1 in case of nonporous composite and $1\text{-}v_f$ is equal to 1 in case of porous matrix ($z \sim z_{mn}$).

↓ Is it possible to check the equation suitability in case of less complex materials?

Nonporous composites are not available. Each used matrix is avilable with 1-2 values of porosity. This situation does not enable fitting as used for porous composites.

↓ Is it possible to check the utilization of simplified equation by another mean?

Yes, it is possible to calculate b exponent, if the measured values z_{mn} and 1-n (= n_p for porous matrix) are installed in the simplified equation for porous matrix. Slope value z_m is taken from composite fitting. Exponent b is then the only unknown equation member.

↓ Is there possibility to create dependences for b sets (different for different properties)?

Figure S5. Flow chart including the thinking process through the whole research—2nd part.

↓ Is there possibility to create dependences for b sets (different for different properties?

Yes, the obtained dependences correspond to those describing b exponents in composite equations in labelling, but the numbers (d and e) are different.

↓

The obtained equations seems to be useful after the employing of the available data.

↓ Is there any other possibility to connect the structure/properties of materials with their mechanical behavior, when the obtained dependence shape was created by searching instead of accurate physical description typical for models?

It could be possible, if some another suitable structural parameter is available.

↓ Is there any other potentionaly suitable structural parameters?

Yes, there is possible to find more structural parameters equal to 1 for nonporous matrix and equal to 1 or lower for more complex materials.

↓ What do the structural parameters fulfill else?

They have to create a pairs of structural parameters (p_1 and p_2), they could be able to differ porous and nonporous material and also pure matrix and composite and retain the basic equation shape.

$$z_c = z_m \cdot p_1^{\,b} \cdot p_2^{\,c}$$

↓ What does it mean matematically?

It is necessary to find a pair, where:
- for nonporous matrix: both of parameters are equal to 1
- for porous matrix/nonporous composite: one parameter is equal to 1 and the second is lower than 1 or both of them are lower than 1
- porous composite: both of parameters are lower than 1

↓ What are the other potential parameters?

Three another potential structural parameters include:
- 1-n (solid rate of material)
- v_m (volume fraction of matrix)
- n_{pf} (matrix rate in solid rate of material = volume fraction of matrix when the porosity is neglected)

$$v_m = (1 - v_f) \cdot n_p$$

$$n_{pf} = \frac{v_m}{1-(1-v_f)+v_m} = \frac{v_m}{v_f + v_m}$$

↓ Are all new potential strutural parameters pairs useful? If not, why?

No, only pairs including v_m are suitable (not all), because:
- parameters multiplication is equal to 1 for porous matrix (n_{pf}/1-v_f)
- parameters multiplication is equal to 1 for nonporous composite (1-n/n_p)
- first fitting gives low R^2 in comparison with n_p/1-v_f (1-n/1-v_f; n_{pf}/1-n; n_{pf}/v_m)
- too different slopes values (property values of nonporous matrices) in comparison with slighly porous matrices (v_m/1-n; n_{pf}/n_p)

↓

The pairs v_m/n_p and v_m/1-v_f enable good ftting and interpolation of obtained b and c parameters by matrix properties. The equations for nonporous matrices were created too. The results are similar to n_p/1-v_f pair.

$$z_c = z_m \cdot v_m^{\,b} \cdot n_p^{\,c}$$

$$z_c = z_m \cdot v_m^{\,b} \cdot (1 - v_f)^c$$

Figure S6. Flow chart including the thinking process through the whole research—3rd part. It is necessary to add that Equation (3) (valid for 1-component materials in the form telling that exponents are number, not function) is mathematically at the beginning, but mentally is in the end. The results occurring in the literature belonging to 1-component materials were found after the research to add a suitable background. The results

not including models/simulations for porous composites were looked for at the beginning of the research without success.
